# Assessing the operational effectiveness of village health volunteers in Thailand: A structural equation modeling analysis

**DOI:** 10.34172/hpp.025.44378

**Published:** 2025-12-30

**Authors:** Jatuporn Ounprasertsuk, Prakasit Wannapaschaiyong, Araya Tipwong

**Affiliations:** ^1^Department of Medical and Public Health Secretary, College of Allied Health Sciences, Suan Sunandha Rajabhat University, Samut Songkhram, Thailand; ^2^Division of Developmental and Behavioral Pediatrics, Department of Pediatrics, Faculty of Medicine Siriraj Hospital, Mahidol University, Bangkok, Thailand; ^3^Department of Community Health Nursing, College of Nursing and Health, Suan Sunandha Rajabhat University, Samut Songkhram, Thailand

**Keywords:** Community health workers, Health services effectiveness, Job satisfaction, Leadership, Organizational commitment, Structural equation modeling

## Abstract

**Background:**

Village health volunteers (VHVs) play an essential role in Thailand’s primary healthcare system by promoting health, preventing diseases, and ensuring community well-being. However, their operational effectiveness is influenced by several key factors, including transformational leadership, employee commitment, and job satisfaction. This study investigates these relationships and their implications for sustainable community health systems.

**Methods:**

A mixed-methods approach was utilized, combining quantitative data from 280 VHVs across Thailand’s lower central region with qualitative insights gained from focus group discussions (FGDs). Structural Equation Modeling was employed to evaluate the relationships among transformational leadership, employee commitment, job satisfaction, and operational effectiveness.

**Results:**

The findings reveal that transformational leadership significantly predicted employee commitment (β=0.80, *P*<0.001) and job satisfaction (β=0.65, *P*<0.001). Operational effectiveness was significantly predicted by employee commitment (β=0.49, *P*<0.001), transformational leadership (β=0.26, *P*<0.001), and job satisfaction (β=0.13, *P*=0.04). Together, these variables explained 68.0% of the variance in operational effectiveness. A new model termed T-E-J Performance (where T=Transformational Leadership, E=Employee Commitment, and J=Job Satisfaction) has been developed to guide strategic improvements.

**Conclusion:**

To sustain effective community health programs, it is vital to enhance leadership capabilities, improve job satisfaction, and foster commitment among VHVs. These findings offer actionable insights for policymakers to create leadership training programs, enhance incentives, and implement strategies for workforce retention. Strengthening VHV systems can significantly contribute to sustainable healthcare delivery and health equity in Thailand.

## Introduction

 Globally, community-based healthcare models have been widely acknowledged for their effectiveness in enhancing public health outcomes, particularly in developing countries.^1^ The World Health Organization considers community health workers to be an essential component in achieving universal health coverage (UHC).^2^ Several Asian nations, including India, Bangladesh, and Indonesia, have introduced community health volunteer programs aimed at extending healthcare services to rural populations. In Thailand, village health volunteers (VHVs) have played a vital role since 1977 in bridging the divide between formal healthcare services and local communities, significantly contributing to disease prevention, health promotion, and access to primary care.^3^

 The effectiveness of VHVs is influenced by several factors, including leadership, job satisfaction, and their commitment to their roles.^4^ Transformational leadership plays a crucial role in shaping the motivation and engagement of VHVs. Additionally, job satisfaction among VHVs significantly affects their retention and performance, while their commitment is essential for operational effectiveness. Understanding these dynamics is vital for developing policies and interventions that enhance the efficiency and sustainability of the VHV program.^5^

 Thailand’s commitment to UHC has heavily relied on the contributions of VHVs, especially in rural areas where medical infrastructure is limited.^6^ VHVs are crucial in delivering maternal and child healthcare, vaccinations, and managing chronic diseases.^2^ They are responsible for providing health education, assisting in disease surveillance, and supporting community-based interventions, thereby ensuring that healthcare services remain accessible, even in remote areas.^7^

 Their responsibilities extend beyond basic healthcare delivery to include social and psychological support, particularly for the elderly and individuals with chronic conditions.^8^ The rising incidence of non-communicable diseases (NCDs) and the need for preventative healthcare measures further underscore the significance of VHVs in Thailand’s healthcare landscape.^9,10^ According to the Thai Ministry of Public Health, NCDs account for approximately 74% of all deaths nationwide, underscoring the need for stronger preventative care systems.^11^

 Leadership—especially transformational leadership—has been shown to shape motivation, innovation, and team engagement.^12^ Similarly, job satisfaction, which encompasses both intrinsic and extrinsic factors, has a significant influence on VHVs’ willingness to continue their service and strive for performance improvement.^13^ Additionally, commitment—whether affective, continuance, or normative—directly impacts the overall operational effectiveness of VHVs.^14^ A strong commitment level ensures that VHVs remain devoted to their responsibilities, thereby reducing turnover rates and improving healthcare outcomes within the community.^15^ While past studies have assessed these factors individually,^16,17^ few have provided a comprehensive model that captures their interrelated impacts on operational effectiveness.^18-20^

 This study introduces the T-E-J Performance Model, which integrates Transformational leadership (T), Employee commitment (E), and Job satisfaction (J) to evaluate VHV’s operational effectiveness. In contrast to previous unidimensional frameworks, this model offers a more holistic, practical, and evidence-based approach. It not only identifies key determinants of VHV performance but also provides actionable insights for strengthening sustainable community health systems.

 The objectives of this study are to: (1) assess the influence of transformational leadership on VHVs’ job satisfaction and commitment, (2) examine the impact of job satisfaction on commitment and operational effectiveness, and (3) develop a structured model to inform policy and intervention strategies for VHVs. The findings of this study could offer valuable insights for policymakers seeking to enhance VHV training and support systems, contribute to the literature on community health volunteer effectiveness, and provide practical recommendations for improving the sustainability of VHV programs in Thailand and other developing countries.

## Methods

###  Study Design 

**Figure 1:**
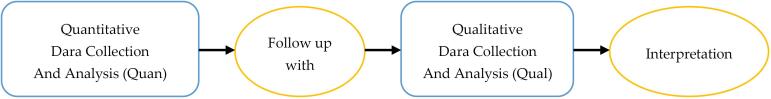
Adapted sequential mixed methods research design with emphasis on quantitative research

 This study employed a mixed-methods research design, combining both quantitative and qualitative approaches. A sequential explanatory strategy was implemented (see Figure 1), where the quantitative phase was conducted first, followed by a qualitative phase aimed at further contextualizing and elucidating the statistical results. This design was selected because it enhances the quantitative findings with in-depth qualitative insights, providing a more comprehensive understanding of the factors affecting operational effectiveness among VHVs. The qualitative data served to clarify and interpret key trends, supporting triangulation and enhancing the validity of the overall findings.

###  Study Population and Sampling

 The target population consisted of 59,817 VHVs located across eight provinces in Thailand’s lower central region: Kanchanaburi, Nakhon Pathom, Ratchaburi, Suphan Buri, Prachuap Khiri Khan, Phetchaburi, Samut Songkhram, and Samut Sakhon. A stratified random sampling method was employed to ensure adequate representation from various provincial health offices. The sample size was determined in accordance with Hair et al^21^ guideline, which recommends 15-20 observations for each observable variable. Given that there were 14 variables, a minimum of 280 participants was necessary to achieve statistical reliability.

 For the qualitative phase, 18 key informants were purposively selected for in-depth focus group discussions (FGDs). The number of participants was determined based on the principle of data saturation, with three FGDs conducted (6 participants per group) until no new themes emerged. Each FGD was facilitated by a trained moderator and an assistant moderator, both with public health research experience, ensuring consistency and reflexivity in data collection.

###  Research Instruments

####  Quantitative Data Collection

 All structured questionnaires were developed in Thai, based on validated tools, and underwent a comprehensive cultural adaptation process. This included forward-translation, expert panel review, and back-translation to ensure semantic and conceptual equivalence. Cognitive interviews with 10 VHVs were conducted during pilot testing to refine item clarity and cultural appropriateness. Each questionnaire demonstrated strong psychometric properties, with content validity index (CVI) values above 0.86 and Cronbach’s alpha coefficients ranging from 0.90 to 0.96, confirming internal consistency. The questionnaire included the following sections:

 Demographic characteristics were developed to record background data, including gender, age, marital status, education, occupation, income, and residence.

 The transformational leadership questionnaire, adapted Khuneepong,^22^ comprises 20 items that focus on four key dimensions: idealized influence, inspirational motivation, intellectual stimulation, and individualized consideration. Participants rated each item on a scale from 1 (strongly disagree) to 5 (strongly agree), resulting in total scores ranging from 20 to 100. Higher scores indicate a greater degree of transformational leadership. The content validity of the questionnaire was assessed by three public health experts, yielding an average item-objective congruence (IOC) value of 0.92. Additionally, its reliability, as measured by Cronbach’s alpha, was established at 0.94.

 The employee commitment questionnaire, adapted from Anunthawichak et al^23^ comprised 18 items that assessed levels of affective, continuance, and normative commitment. Respondents rated each item on a scale from 1 (strongly disagree) to 5 (strongly agree), resulting in total scores that ranged from 18 to 90. Higher scores indicate greater employee commitment. The content validity of the questionnaire was assessed by three public health experts, who yielded an average IOC value of 0.90. Additionally, the reliability, measured by Cronbach’s alpha, was determined to be 0.94.

 The job satisfaction questionnaire, adapted from Bunnag et al^24^ consists of 20 items that address two key dimensions: intrinsic and extrinsic motivation factors. Participants rated each item on a scale from 1 (strongly disagree) to 5 (strongly agree), resulting in total scores that range from 20 to 100. A higher score indicates a greater level of job satisfaction. The content validity of the questionnaire was evaluated by three public health experts, who determined an average IOC value of 0.96. Furthermore, the reliability of the questionnaire, assessed using Cronbach’s alpha, was found to be 0.90.

 The operational effectiveness questionnaire, adapted from Akasriworn^25^ consisted of 12 items addressing health promotion, disease prevention, rehabilitation, consumer protection, and community health management. Respondents rated each item on a scale from 1 (strongly disagree) to 5 (strongly agree), resulting in total scores ranging from 12 to 60. Higher scores indicate greater operational effectiveness. The questionnaire’s content validity was evaluated by three public health experts, yielding an average IOC value of 0.86, while the reliability, measured by Cronbach’s alpha, was found to be 0.96.

####  Qualitative Data Collection

 The qualitative phase comprised three FGDs with 18 VHVs. A semi-structured interview guide was used to explore themes related to leadership, job satisfaction, and operational effectiveness. FGDs were audio-recorded, transcribed verbatim, and analyzed using thematic content analysis supported by NVivo software. The moderator guided discussions to ensure depth while the assistant took field notes and facilitated follow-up questions.

###  Data Collection 

 Quantitative data collection took place from March to May 2024, utilizing both paper-based and online surveys, which were administered by provincial public health coordinators. A total of 320 questionnaires were distributed, yielding 280 valid responses (response rate: 87.5%). The qualitative FGDs were conducted in June 2024, with each session lasting 60-90 minutes. Participants were encouraged to share their experiences regarding leadership, job satisfaction, and their overall role in the healthcare system.

###  Data Integration

 Integration of quantitative and qualitative findings occurred during the interpretation phase. Themes emerging from the FGDs were mapped onto the quantitative results to identify convergence, divergence, and complementarity. For instance, qualitative insights were used to explain why certain variables had stronger effects in the SEM model, enhancing the interpretation of causal pathways. This triangulation strengthened the trustworthiness and applicability of the study’s conclusions.

###  Data Analysis

####  Quantitative Analysis

 Descriptive Statistics were employed to summarize the demographic characteristics of the sample. Inferential Statistics were utilized to test the research hypotheses. IBM SPSS Statistics (Version 26; IBM Corp., Armonk, NY, USA) was used to assess relationships among variables. Structural Equation Modeling (SEM) was conducted using SmartPLS (Version 4.0; SmartPLS GmbH, Oststeinbek, Germany) to evaluate the hypothesized relationships among latent variables using a partial least squares structural equation modeling (PLS-SEM) approach. PLS-SEM was selected due to its suitability for prediction-oriented models and complex relationships involving multiple latent constructs.^26^ Prior to conducting the analysis, all relevant assumptions were confirmed to be met. Model evaluation followed established PLS-SEM guidelines, including assessment of path coefficients, coefficient of determination (R^2^), and significance testing using bootstrapping procedures. Model fit was assessed using the standardized root mean square residual (SRMR), with values ≤ 0.08 indicating acceptable fit. The statistical significance level for all analyses was set at *P* < 0.05.

####  Qualitative Analysis

 In this study, we implemented a thematic analysis approach to systematically identify and examine recurring patterns within the participants’ responses. To support this process, we utilized NVivo software, which offered robust tools for coding data and establishing a clear thematic framework. This enabled us to explore the nuances of the responses in depth, uncovering significant insights and underlying themes throughout the qualitative data.

## Results

###  Characteristics of Participants 

 The study sample comprised 280 VHVs from eight provinces in Thailand’s lower central region. The findings indicate that a significant majority of the VHVs in this study were female (65.71%), with the largest age group being 31-40 years old (42.86%), and many were married (51.8%). Most participants had completed either primary education (40.00%) or were primarily engaged in agriculture (43.21%). Additionally, a considerable portion of the participants (31.79%) reported a monthly income of less than 5,000 Baht, while 68.21% owned their homes (see Table 1).

**Table 1:** Demographic and socioeconomic characteristics of village health volunteers (n = 280)

**Characteristic**	**Category**	**n**	**%**
Gender	Male	96	34.29
	Female	184	65.71
Age group (years)	< 30	79	28.21
	31-40	120	42.86
	41-50	48	17.14
	> 50	33	11.79
Marital status	Married	145	51.79
	Single	131	46.79
	Widowed/Divorced/Separated	4	1.43
Education level	Primary education	112	40.00
	Secondary education	109	38.93
	Bachelor's degree	57	20.36
	Master’s degree	2	0.71
Occupation	Agriculture	121	43.21
	General labor	84	30.00
	Trading	31	11.07
	Government service	29	10.36
	Others	15	5.36
Monthly income (Baht)	< 5,000	89	31.79
	5,001-10,000	68	24.29
	10,001-15,000	53	18.93
	15,001-20,000	43	15.36
	> 20,000	27	9.64
Residence	Own House	191	68.21
	Rental House	58	20.72
	Living with relatives	31	11.07

 The findings reveal that VHVs hold a high perception of transformational leadership, employee commitment, job satisfaction, and operational effectiveness, with all mean scores surpassing 3.70. Operational effectiveness received the highest rating (M = 3.81, SD = 0.69), particularly within health rehabilitation (M = 3.89, SD = .88), underscoring the crucial role of VHVs in patient care and recovery. Employee commitment also scored highly (M = 3.76, SD = 0.65), with continuance commitment being the strongest aspect (M = 3.83, SD = 0.69), indicating that VHVs are motivated to remain in their positions due to job stability and a strong sense of responsibility. Transformational leadership was positively evaluated (M = 3.78, SD = 0.74), although intellectual stimulation received the lowest score (M = 3.66, SD = 0.77), suggesting a need for enhanced encouragement of innovation and problem-solving. Job satisfaction was reported at (M = 3.73), revealing that VHVs are more driven by intrinsic factors (M = 3.77) than extrinsic rewards (M = 3.70), highlighting their deep personal commitment to their work (see Table 2).

**Table 2:** Mean, standard deviation (SD), and interpretation of transformational leadership, employee commitment, job satisfaction, and operational effectiveness among VHVs

**Variables**	**Mean**	**SD**	**Interpretation**
Transformational leadership (TRLE)	3.78	0.74	High
Idealized influence (IDIN)	3.78	0.74	High
Inspirational motivation (INSP)	3.74	0.80	High
Intellectual stimulation (INST)	3.66	0.77	High
Individual consideration (COIN)	3.73	0.78	High
Employee commitment (EMEN)	3.76	0.65	High
Affective commitment (EMCO)	3.67	0.76	High
Continuance commitment (COEN)	3.83	0.69	High
Normative commitment (NOCO)	3.80	0.71	High
Job satisfaction (JOSA)	3.73	0.61	High
Intrinsic motivation factors (INMO)	3.77	0.65	High
Extrinsic motivation factors (EXMO)	3.70	0.66	High
Operational effectiveness (OPEF)	3.81	0.69	High
Health promotion (HEPR)	3.74	0.80	High
Disease surveillance, prevention, and control (DSPC)	3.79	0.73	High
Health rehabilitation (HERE)	3.89	0.88	High
Consumer protection (COPR)	3.83	0.83	High
Community health management and sub-district health plan participation (CHMP)	3.81	0.82	High

 The conceptual framework of this research is organized around four latent variables and fourteen observable indicators. These latent variables encompass transformational leadership (TRLE), employee commitment (EMEN), job satisfaction (JOSA), and operational effectiveness (OPEF). This framework aims to elucidate the relationships among these latent constructs and their corresponding measurable elements, offering a comprehensive understanding of the factors that influence VHV performance.

**Figure 2:**
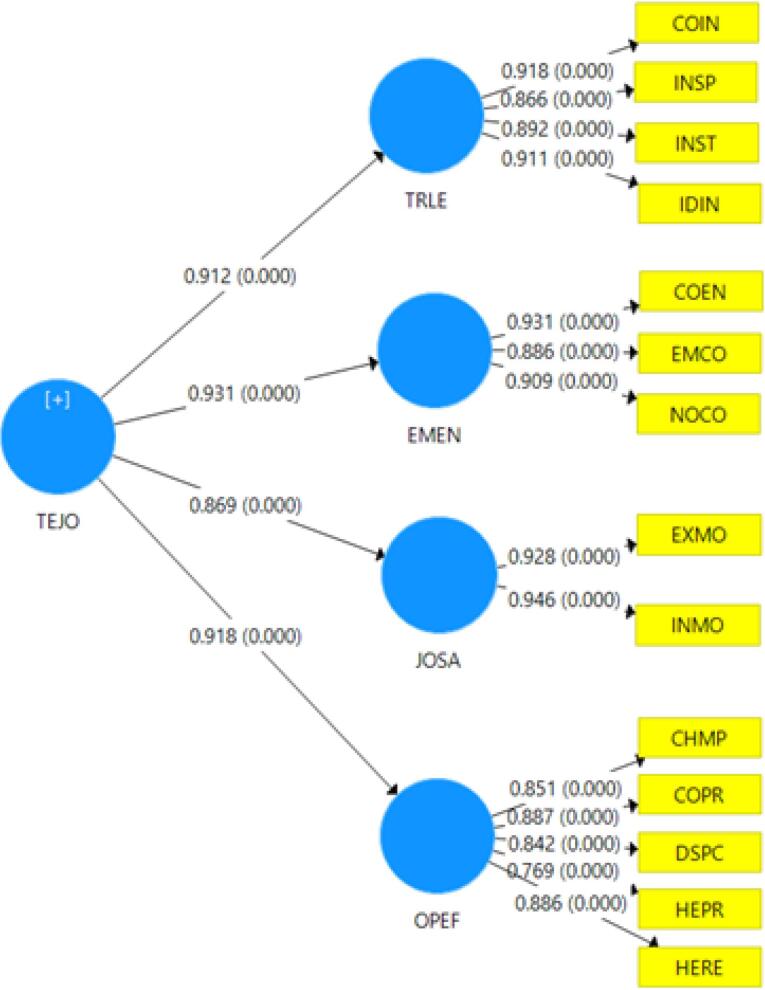
Measurement model evaluation results using confirmatory factor analysis (CFA) for latent variables in the structural equation model (PLS-SEM)

 As illustrated in Figure 2, the latent variables are represented by ellipses, while the observable variables are depicted as rectangles. This structural representation is consistent with the confirmatory factor analysis (CFA) approach employed in PLS-SEM to validate the relationships among the constructs. The diagram effectively demonstrates the interconnections between TRLE, EMEN, JOSA, and OPEF, along with their respective observable indicators, thereby providing clarity in assessing how each latent variable contributes to overall operational effectiveness.

 The SEM analysis evaluates the relationships between TRLE, EMEN, and JOSA and their direct and indirect effects on the OPEF of VHVs in Thailand’s Lower Central Region. This model provides empirical insights into the key factors influencing the efficiency and effectiveness of VHVs in delivering healthcare services.

**Figure 3:**
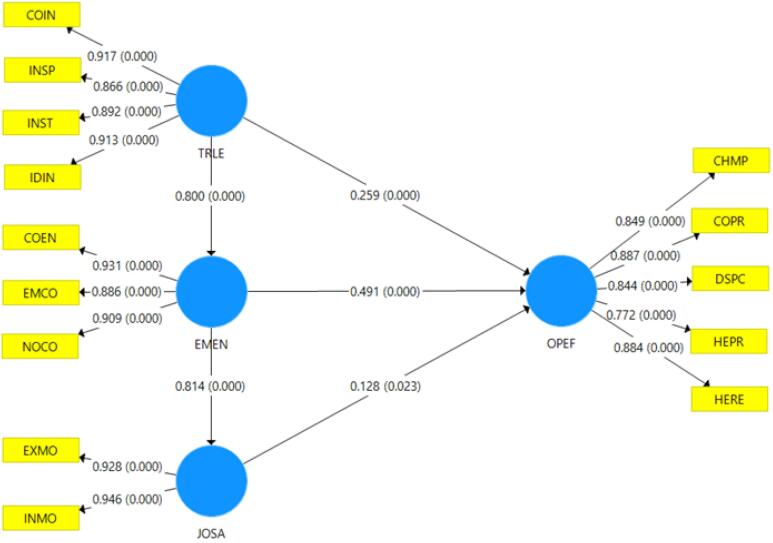
Results of statistical significance testing using bootstrapping

 Additionally, 14 observable variables were assessed, each demonstrating outer loading values above 0.70, meeting the threshold for construct validity and reliability. These validated constructs were then analyzed using PLS-SEM, as illustrated in Figure 3. TRLE plays a pivotal role in enhancing OPEF by providing guidance, motivation, and strategic direction for VHVs. EMEN significantly influences OPEF by fostering VHV retention, engagement, and dedication to healthcare services. Meanwhile, JOSA is crucial in shaping VHV morale, motivation, and their overall willingness to perform effectively in community health settings. These findings underscore the interconnected relationships among leadership, commitment, satisfaction, and operational performance, highlighting the necessity for stronger leadership structures, increased workforce engagement, and improved support mechanisms to maintain VHV effectiveness within Thailand’s healthcare system. This demographic distribution illustrates the typical profile of VHVs in Thailand, emphasizing their vital role as community-based healthcare facilitators in rural and semi-urban areas.

 The SEM analysis showed that TRLE, EMEN, and JOSA collectively explained 68.0% of the variance in operational effectiveness (OPEF), indicating a substantial influence on VHV performance. Employee commitment exerted the strongest direct effect on OPEF (β = 0.49, *P* < 0.001), followed by transformational leadership (β = 0.26, *P* < 0.001) and job satisfaction (β = 0.13, *P* < 0.05) (Table 3).

**Table 3:** Parameter estimation results of direct effects, indirect effects, and total effects from the adjusted structural equation model

**Dependent variable**	**R^2^**	**Effect Type**	**Independent Variables**		
			**Transformational leadership (TRLE)**	**Employee commitment (EMEN)**	**Job satisfaction (JOSA)**
Employee commitment (EMEN)	0.64	Direct	0.80*** (30.36)	-	-
		Indirect	-	-	-
		Total	0.80*** (30.36)	-	-
Job satisfaction (JOSA)	0.66	Direct	-	0.81*** (32.13)	-
		Indirect	0.65*** (17.71)	-	-
		Total	0.65*** (17.71)	0.81*** (32.13)	-
Operational effectiveness (OPEF)	0.68	Direct	0.26*** (3.56)	0.49*** (5.79)	0.13* (2.04)
		Indirect	0.48*** (7.98)	0.10* (2.01)	-
		Total	0.74*** (21.29)	0.60*** (8.61)	0.13* (2.04)

Note: *Significant at.05 level (*P* < 0.05), **Significant at.01 level (*P* < 0.01), ***Significant at.001 level (*P* < 0.001).

 Additionally, the indirect effects analysis suggests that TRLE and EMEN also influence OPEF through intermediary pathways, further reinforcing their role in enhancing VHVs’ performance. The total effect values of TRLE (0.735), EMEN (0.595), and JOSA (0.128) highlight that while all three factors contribute to OPEF, EMEN serves as a critical mediator in improving operational effectiveness.

 The in-depth interviews revealed that the operational effectiveness of VHVs is significantly influenced by their knowledge, experience, training, and initiatives aimed at building confidence. Among the key factors identified, TRLE emerged as the most frequently discussed variable, as it cultivates a positive work ethos, motivation, and job satisfaction. VHVs noted that effective leadership enhances their organizational engagement, administrative efficiency, interpersonal relationships, working conditions, job security, salary, benefits, professional status, and interactions with supervisors.

 A high level of JOSA proved to be a critical determinant of EMEN, ensuring that VHVs remain dedicated to their responsibilities and strive for optimal performance. The interplay among these factors underscores the necessity for a structured approach to improving VHV effectiveness.

**Figure 4:**
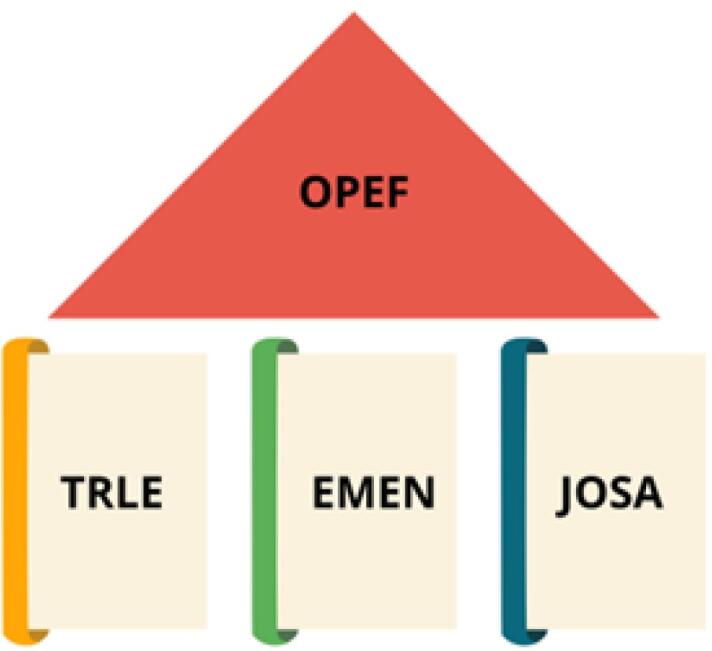
“T-E-J performance” model. Note: TRLE = Transformational Leadership, EMEN = Employee Commitment, JOSA = Job Satisfaction, OPEF = Operational Effectiveness)

 In light of these findings, the researchers developed the “T-E-J Performance Model,” which serves as a practical framework for administrators and policymakers to optimize the quality of work, motivation, and overall effectiveness of VHVs. This model illustrates that TRLE, EMEN, and JOSA serve as foundational pillars supporting OPEF, as depicted in Figure 4.

## Discussion

 The operational effectiveness of VHVs in Thailand represents a complex and multifaceted construct that integrates five essential components: health promotion, disease surveillance and prevention, health rehabilitation, consumer protection, and community health management, all actively engaging in sub-district health plans.^3^ Together, these components contribute to improved community health outcomes, highlighting the crucial role that VHVs play in strengthening primary healthcare systems in resource-limited settings.^6^

 This study provides empirical evidence indicating that transformational leadership, employee commitment, and job satisfaction have a significant influence on VHV performance. Structural equation modeling revealed that employee commitment had the strongest direct effect, followed by transformational leadership and job satisfaction. Qualitative findings reinforced these quantitative results. For example, one VHV stated, “*When our leaders support us, we feel more motivated to stay and serve our community with pride*,” while another shared, “*It’s not the money that keeps us here, it’s the feeling that we matter*.” These insights reflect how intrinsic rewards and supportive leadership foster sustained engagement.

 The findings presented hold significant implications for policymakers, healthcare administrators, and community health programs. It is essential to implement leadership development initiatives that foster transformational leadership among VHV coordinators, with an emphasis on mentorship, effective communication, and motivational strategies. Furthermore, establishing targeted incentive structures—such as community recognition programs, opportunities for career advancement, and modest financial support—can enhance employee commitment and decrease turnover rates. Enhancing workplace conditions by providing sufficient resources, peer support systems, and access to mental health services will further improve job satisfaction and encourage long-term retention. These strategies align with national objectives aimed at increasing access to primary healthcare and addressing the growing burden of NCDs in Thailand.

 Transformational leadership is a pivotal factor in the operational effectiveness of VHVs, influencing their motivation, engagement, and performance. This leadership style is characterized by four key dimensions: idealized influence, inspirational motivation, intellectual stimulation, and individualized consideration. Leaders who demonstrate idealized influence act as role models, cultivating trust, credibility, and a shared vision among VHVs.^27,28^ Inspirational motivation encourages volunteers to connect with a compelling mission, thereby enhancing their commitment to public health service.^27^ Intellectual stimulation fosters innovative solutions to health challenges, promoting problem-solving skills and adaptability among VHVs.^28^ Finally, individualized consideration addresses the unique needs and aspirations of VHVs, contributing to increased job satisfaction and retention.^14,29^ Empirical research has established a strong positive correlation between transformational leadership and operational effectiveness in various community health programs.^28^ Consequently, enhancing leadership capabilities within VHV networks may serve as a strategic intervention to improve healthcare delivery and sustainability.^6,30^

 Employee commitment serves as a fundamental pillar of VHV performance, encompassing three critical dimensions: affective commitment, continuance commitment, and normative commitment. Affective commitment pertains to an individual’s emotional attachment to their role, which cultivates loyalty, engagement, and intrinsic motivation.^31^ Continuance commitment is linked to the perceived costs of leaving the organization, thereby reinforcing volunteer retention and the sustainability of roles.^32^ Normative commitment arises from moral and social obligations, motivating VHVs to remain dedicated to their responsibilities and community service.^32^ A strong commitment among VHVs enhances service quality, program efficiency, and long-term sustainability.^33,34^ Research has consistently shown that higher levels of employee commitment are associated with improved healthcare outcomes and increased community trust in healthcare systems.^28,35^

 Job satisfaction plays a crucial role in influencing engagement, retention, and effectiveness among VHVs, making it a vital component for sustaining high-performing volunteer networks.^13^ This construct is fueled by both extrinsic and intrinsic motivators. Extrinsic factors encompass organizational policies, financial compensation, workplace conditions, job security, and supervisory relationships.^36^ Conversely, intrinsic factors include work achievement, recognition, job autonomy, responsibility, and opportunities for career advancement.^37^ Research consistently demonstrates that greater job satisfaction correlates with increased productivity, lower absenteeism, and enhanced service delivery among VHVs.^38^ Furthermore, job satisfaction is a significant determinant of volunteer retention, underscoring the importance of supportive organizational frameworks that foster professional fulfillment and motivation.^39^

 Compared to prior research, which often focuses on isolated variables such as training or workload, this study proposes a holistic model—the T-E-J Performance Model—that integrates leadership, commitment, and job satisfaction. This model explained 68% of the variance in operational effectiveness, offering more robust predictive utility than unidimensional frameworks. For instance, while a prior study emphasized peer training outcomes, they did not account for leadership dynamics or motivational constructs.^5^ Our integrated approach provides a practical tool for designing community-based interventions and training strategies.

 This study offers valuable insights into the factors that influence the operational effectiveness of VHVs, but it is important to acknowledge several limitations. First, the research was conducted within a specific geographic region in Thailand, which may restrict the generalizability of the findings to other contexts that have different healthcare infrastructures and community engagement models. Future research should investigate these relationships in a variety of geographic and socio-cultural settings to improve external validity. Second, the study utilized a cross-sectional design, capturing data at a single moment in time, which limits the ability to establish causal relationships among transformational leadership, commitment, job satisfaction, and operational effectiveness. Adopting a longitudinal approach could enhance our understanding of how these variables may change over time. Third, while self-reported data were obtained through structured questionnaires, there is a possibility that social desirability bias influenced the responses. Future studies should consider employing a mix of data collection methods, including qualitative interviews and direct observations, to validate the findings. Finally, this study concentrated on organizational and individual-level factors affecting VHV effectiveness but did not thoroughly explore external influences such as government policies, funding mechanisms, and systemic health barriers. Further research should investigate these macro-level determinants to develop more comprehensive intervention strategies.

 The generalizability of these findings should be interpreted with caution. This study was conducted among VHVs in Thailand’s lower central region, where organizational structures, training systems, and sociocultural contexts may differ from other regions or countries. As a result, the findings may not be directly generalizable to all community health worker programs or healthcare systems with different governance, incentive structures, or workforce characteristics.

 Nevertheless, the theoretical relationships identified in this study, particularly the roles of transformational leadership, employee commitment, and job satisfaction in shaping operational effectiveness, are grounded in well-established organizational and behavioral theories. These mechanisms are likely to be relevant to community health workers and volunteer-based health systems in similar low- and middle-income settings. Future studies employing longitudinal designs, multi-region samples, or cross-country comparisons are recommended to assess further the external validity and broader applicability of the T-E-J Performance Model.

## Conclusion

 This study emphasizes the critical role of VHVs in enhancing community-based healthcare services in Thailand. By exploring the relationships among transformational leadership, employee commitment, job satisfaction, and operational effectiveness, the findings highlight the significance of leadership development, workforce engagement, and job satisfaction in optimizing VHV performance. The results indicate that transformational leadership promotes motivation, trust, and innovation, ultimately resulting in higher levels of commitment and job satisfaction. Furthermore, employee commitment is a key factor in VHV retention and performance, underscoring the necessity for organizational policies that foster long-term engagement. Job satisfaction, influenced by both intrinsic and extrinsic factors, directly affects volunteer efficiency and effectiveness, reinforcing its essential role in the sustainability of VHVs. For policymakers and healthcare administrators, this study offers actionable insights for enhancing VHV training programs, leadership development initiatives, and strategies for workforce retention. By addressing these aspects, stakeholders can improve the efficiency of VHV programs, strengthen community health systems, and contribute to the overarching goal of universal healthcare coverage in Thailand. Future research should investigate longitudinal studies to evaluate the long-term effects of leadership and commitment on VHV effectiveness, as well as explore additional contextual factors, such as policy frameworks and funding mechanisms, that affect VHV sustainability. Ultimately, the T-E-J Performance Model not only offers actionable insights for Thailand’s community health system but also serves as a transferable framework for countries seeking to strengthen their volunteer-based healthcare infrastructure and promote global health equity.

## Competing Interests

 The authors declare no conflicts of interest.

## Data Availability Statement

 The data sets of this study are not publicly available due to the information that could compromise the research participants’ privacy. However, data may be shared upon request by the authors.

## Ethical Approval

 The study was conducted in accordance with the Declaration of Helsinki, and approved by the Institutional Review Board of the Research Ethics Committee of the Research and Development Institute, Suan Sunandha Rajabhat University (protocol code COA.0-021/2027 and dated 14 May 2024).
